# Association of Essential Tremor With Dementia and Affective Disorders: A Meta-Analysis

**DOI:** 10.3389/fneur.2022.842732

**Published:** 2022-03-17

**Authors:** Yajun Shang, Xinjie Chen, Mingda Ai, Xiaoran Gao, Shujuan Dai, Mingjie Zhao, Cen Yang, Liangfeng Wang, Junyan Zhang, Lianmei Zhong, Tianhao Bao, Xiaolei Liu

**Affiliations:** ^1^Department of Neurosurgery, The First Affiliated Hospital of Kunming Medical University, Kunming, China; ^2^Yunnan Provincial Clinical Research Center for Neurological Diseases, Kunming, China; ^3^Department of Neurology, The First Affiliated Hospital of Dali University, Dali, China; ^4^Department of Neurology, The First Affiliated Hospital of Kunming Medical University, Kunming, China; ^5^Department of Surgery, Shenzhen University, Shenzhen, China; ^6^Department of Anesthesiology, The First Clinical Medical College of Kunming Medical University, Kunming, China; ^7^Bothwin Clinical Study Consultant, Shanghai, China; ^8^Department of Geriatrics, Kunming Medical University Affiliated Mental Health Center, Kunming, China; ^9^West China Hospital, Sichuan University, Chengdu, China

**Keywords:** anxiety, dementia, essential tremor, depression, movement disorders

## Abstract

**Background:**

The dementia and affective disorders are common non-motor features in patients with essential tremor (ET). However, the relationship of ET with cognitive impairments and affective disorders remains controversial. This meta-analysis aimed to analyze the association of ET with dementia and affective disorders.

**Methods:**

Original studies published from January 1999 to October 2019 were systematically searched from the database of Medline (OvidSP), EMBASE (OvidSP), and the Cochrane Central Register of Controlled Trials. Pooled standard mean difference (SMD, random effect model), odds ratios (ORs), relative risk (RR), and 95% *CI* were calculated.

**Results:**

Compared with the Non-ET group, patients with ET had significantly lower Mini-Mental State Examination (MMSE) score (*SMD*, −1.16; *95% CI*, −1.75 to −0.58; *p* = 0.0001) and had significantly higher depressive and anxiety symptoms scale score (*SMD*, 0.55; *95% CI*, 0.22–0.87; *p* = 0.0009). The OR for dementia and affective disorders in individuals with ET compared with individuals without ET was 2.49 (*95% CI*, 2.17–2.85, *p* < 0.00001). While there was no significant difference in Montreal Cognitive Assessment (MoCA) score between ET and Non-ET groups (*SMD*, −0.52; *95% CI*, −0.16 to 0.13; *p* = 0.23), there was a significant difference in the risk of mortality between ET and Non-ET groups (*RR* = 4.69, *95% CI*, 2.18–10.07).

**Conclusion:**

The non-motor symptoms should not be neglected among patients with ET. However, the causal relationship between ET and dementia, depression, and anxiety is unclear.

## Introduction

Essential tremor (ET) is one of the common movement disorders and is characterized by isolated tremor syndrome of the bilateral upper limbs for at least 3 years. The prevalence of ET is about 5% in the general population and over 20% in the elderly (1, 2). ET is often familial, with a typically autosomal dominant pattern, and is most often observed in the elderly population over 65 years old. The main clinical manifestations of ET patients include motor features (intention tremor and ataxia) and non-motor features (cognitive impairments and affective disorders). ET often has a bilateral presentation but can also be asymmetric in nature and is rarely seen at rest. Tremor is exaggerated under stressful conditions, and 50–70% of ET cases can be ameliorated by consuming alcohol (3).

There is an increasing awareness that patients with ET may present non-motor features, such as cognitive impairments and mood disorders. In 2001, it was suggested that tremor was not the only manifestation of ET. Since then, a rising number of non-motor symptoms were recognized in patients with ET, such as not only cognitive impairment and affective disorders but also anxiety, personality changes, fatigue, hearing impairment, olfactory dysfunction, upper airway dysfunction, and sleep disturbances (4, 5). Meanwhile, the cognitive deficits of ET were first noticed prior to thalamic deep-brain stimulation (DBS) for refractory ET (6). Cognitive deficits were observed in 69.2% of patients with ET, and 11.4% of ET had dementia. The cognitive deficits affected the domains of attention, executive function, and memory (7).

An increasing number of studies have shown the poor quality of life among patients with ET. Compared to physical impairments, psychological and cognitive deficits more likely affected patients with ET because of the psychological burden and negative influence on the recovery (4). Therefore, it is necessary to better understand the correlation of ET with cognitive impairment and affective disorders. This meta-analysis aimed to analyze the correlation of ET with dementia and affective disorders.

## Methods

### Search Strategy

This meta-analysis followed the Meta-analysis Of Observational Studies in Epidemiology (MOOSE) group reporting guideline (Supplementary File S1) (8), and the Preferred Reporting Items for Systematic reviews and Meta-Analyses (PRISMA) guidelines (Supplementary File S2). The literature search, title screening, abstract screening, final decision on eligibility after full-text review, methodological quality appraisal of included studies, and data extraction were independently performed by two investigators.

Original studies published from January 1999 to October 2019 were systematically searched from the database of Medline (OvidSP), EMBASE (OvidSP), and the Cochrane Central Register of Controlled Trials (CENTRAL, last issue). Searching terms were listed as follows:

1) Essential Tremor.
2) (essential tremor or Familial Tremor).mp.
3) 1 OR 2.
4) (observational or “case control” or cohort or “cross sectional” or “follow up” or risk factor^*^).mp.
5) 3 AND 4.

Publication type and language were not restricted in the searches. Reference lists of all relevant articles were also searched for additional studies.

### Inclusion and Exclusion Criteria

Identified articles were then further selected if they satisfied all of the following conditions:

1) Compared ET participants with healthy control.
2) The age of participants with more than 18 years.
3) Studies provided either the Montreal Cognitive Assessment (MoCA), Mini-Mental State Examination (MMSE) score, and Hamilton Anxiety Rating Scale (HARS), Hamilton Depression Rating Scale (HDRS) score, Beck Depression Inventory (BDI), or the association (when a reference comparison group was available) of dementia or anxiety, depression.
4) Published in English only.

Searched articles that met one of the following criteria were excluded:

1) Cannot find the original paper.
2) Data cannot be extracted.
3) Authors did not use mean (SD) report continuous outcomes or did not report event numbers for dementia or anxiety, depression, or did not report outcomes with OR, HR, RR together with 95% CI.
4) The participants with nervous and psychiatric system diseases seriously affecting cognitive function and emotion.

### Study Selection and Data Extraction

In the initial screening, two authors (Xinjie Chen and Mingda Ai) independently assessed all of the abstracts retrieved from the search and excluded studies that met the exclusion criteria. Then full manuscripts of the potentially eligible studies were obtained and screened twice for inclusion.

A third author (Xiaolei Liu) solved any disagreements based on the criteria of inclusion and exclusion. In addition, the following variables were extracted when available (shown in Table 1), i.e., (1) author; (2) year of published; (3) region; (4) age of participants; (5) educational status of participants; (6) age at onset for tremor; (7) duration of illness; (8) method used to assess cognitive study or depression or anxiety (i.e., structured diagnostic interview or coded diagnosis given by clinician or screening tool or validated algorithm); (9) study design; and (10) follow-up period.

**Table 1:** Characteristics of included studies.

**References**	**Year**	**Study characteristics**	**Region**	**Number of analyzed ET patients**	**Mean age (year)**	**Sex (female, %)**	**Mean education (years)**	**Mean age at onset (years)**	**Mean duration of illness (years)**	**Method used to assess cognitive impairment or depression or anxiety**	**Exposure sub-groups**	**Mean follow-up (years)**
Dogu et al. (9)	2005	Cross-sectional	Turkey	89	57.3	47.3	3.4	NA	9.5	HDS/HAS	depression/anxiety	NA
Benito-León et al. (10)	2006	Cross-sectional	Spain	232	75	59.5	NA	NA	3	37-MMSE	dementia	NA
Louis et al. (11)	2007	Prospective	Spain	78	73.5	33.6	NA	NA	NA	clinical questionnaire/DSM-IV	depression/dementia	NA
Louis et al. (12)	2007	Prospective	Spain	201	75.1	60.7	NA	NA	NA	NA	mortality	3.2
Kim et al. (13)	2009	Cross-sectional	Korea	34	67.6	26.5	10.7	NA	7.3	K-MMSE	dementia	NA
Thawani et al. (14)	2009	Prospective	Australia	93	NA	NA	NA	NA	NA	DSM-III-R	dementia	3.8
Louis et al. (15)	2010	Prospective	Spain	135	73.6	58.5	NA	NA	NA	37-MMSE clinical questionnaire	dementia/depression	3.4
Louis et al. (16)	2010	Cross-sectional	Spain	208	75.1	59.6	NA	NA	9.6	37-MMSE	dementia	NA
Louis et al. (17)	2011	Cross-sectional	Spain	237	75	59.9	NA	NA	NA	clinical questionnaire	depression	NA
Passamonti et al. (18)	2011	Cross-sectional	NA	15	61.6	33.3	9.8	45	16.6	MMSE HAMA/BDI	dementia/ anxiety/depression	NA
Benito-León et al. (19)	2011	Prospective	Spain	207	76	57	NA	NA	10.2	clinical questionnaire	depression	3.4
Chandran et al. (20)	2012	Cross-sectional	Indian	50	40.7	72	NA	32.2	8.4	HDRS/HARS	depression/anxiety	NA
Cerasa et al. (21)	2014	Cross-sectional	NA	14	66.3	43	NA	53.2	12.8	MMSE	dementia	NA
Rao et al. (22)	2013	Cross-sectional	NA	61	84.4	68	NA	43.3	NA	mMMSE	dementia	NA
Benito-León et al. (23)	2013	Prospective	Spain	56 (premotor ET)	73	57.1	NA	NA	NA	37-MMSE clinical questionnaire	dementia/depression	3.4
Benito-León et al. (23)	2013	Prospective	Spain	135 (prevalent ET)	73.6	59.3	NA	NA	NA	37-MMSE clinical questionnaire	dementia/depression	3.4
Park et al. (24)	2015	Cross-sectional	Korea	45	68.8	22.2	NA	NA	NA	K-MMSE	dementia	NA
Sengul et al. (25)	2016	Cross-sectional	Erzurum	30	27.7	60	NA	NA	5.1	MoCA	dementia	NA
Benito-León et al. (26)	2016	Prospective	Spain	78 (premotor ET)	73.4	57.7	NA	NA	NA	37-MMSE	dementia	3.2
Benito-León et al. (26)	2016	Prospective	Spain	206 (prevalent ET)	74.6	62.6	NA	NA	NA	37-MMSE	dementia	3.2
Meyers et al. (27)	2019	Cross-sectional	American	156	60.1	64.7	16.6	42.3	NA	MoCA	dementia	NA

*NA, Not available from original study paper or supplementary or registration information; HDS, Turkish versions of the Hamilton Depression Rating Scale; HAS, Turkish versions of the Hamilton Anxiety Rating Scale;37-MMSE,37-item version of the Mini-Mental State Examination; HARS, Hamilton Anxiety Rating Scale; HDRS, Hamilton Depression Rating Scale; mMMSE, modified Mini Mental State; k-MMSE, Korea-version of the Mini-Mental State Examination; MoCA, Montreal Cognitive Assessment; prevalent ET cases, participants diagnosed with ET at baseline and at follow-up; premotor ET cases, participants diagnosed with incident ET at follow-up, but not at baseline; DSM, The Diagnostic and Statistical Manual of Mental disorders*.

### Methodological Quality Appraisal

The Agency for Healthcare Research and Quality (AHRQ) was used to rate the methodological quality of cross-sectional studies. An item would be scored “0” if it was answered “NO” or “UNCLEAR” and scored “1” if it was answered “YES”. Article quality was assessed as follows: low quality = 0–3; moderate quality = 4–7; and high quality = 8–11. The quality of studies was assessed using the Newcastle Ottawa Scale (NOS) generating a maximum of nine stars for each study, i.e., four stars for the selection of participants, two stars for the comparability of participants, and three stars for the assessment of outcomes. Quality was assigned according to the scores so that 7–9 stars indicated high quality, 4–6 stars for middle quality, and 0–3 stars for low quality (28).

### Statistical Analysis

All statistical analyses were conducted using statistical software Cochrane Collaboration (RevMan5.3) and Stata15.0. Results were reported as OR with corresponding 95% CI for dichotomous data. The mean difference (MD) or SMD was calculated for continuous data. Transformed into SMD when different scales were used for the same outcome domain. Heterogeneity between study results was assessed using a standard *I2* test, with an *I*^2^ >50% regarded as an indicator of substantial heterogeneity, then the random-effects model will be implemented (29). Publication bias was assessed via funnel plots and more formally with the Begg's test. Potential sources of heterogeneity across studies were explored by subgroup analyses. The following variables were considered: (1) type of disease; (2) study characteristics; and (3) method used to assess cognitive study or depression or anxiety.

## Results

### Study Selection

**Figure 1:**
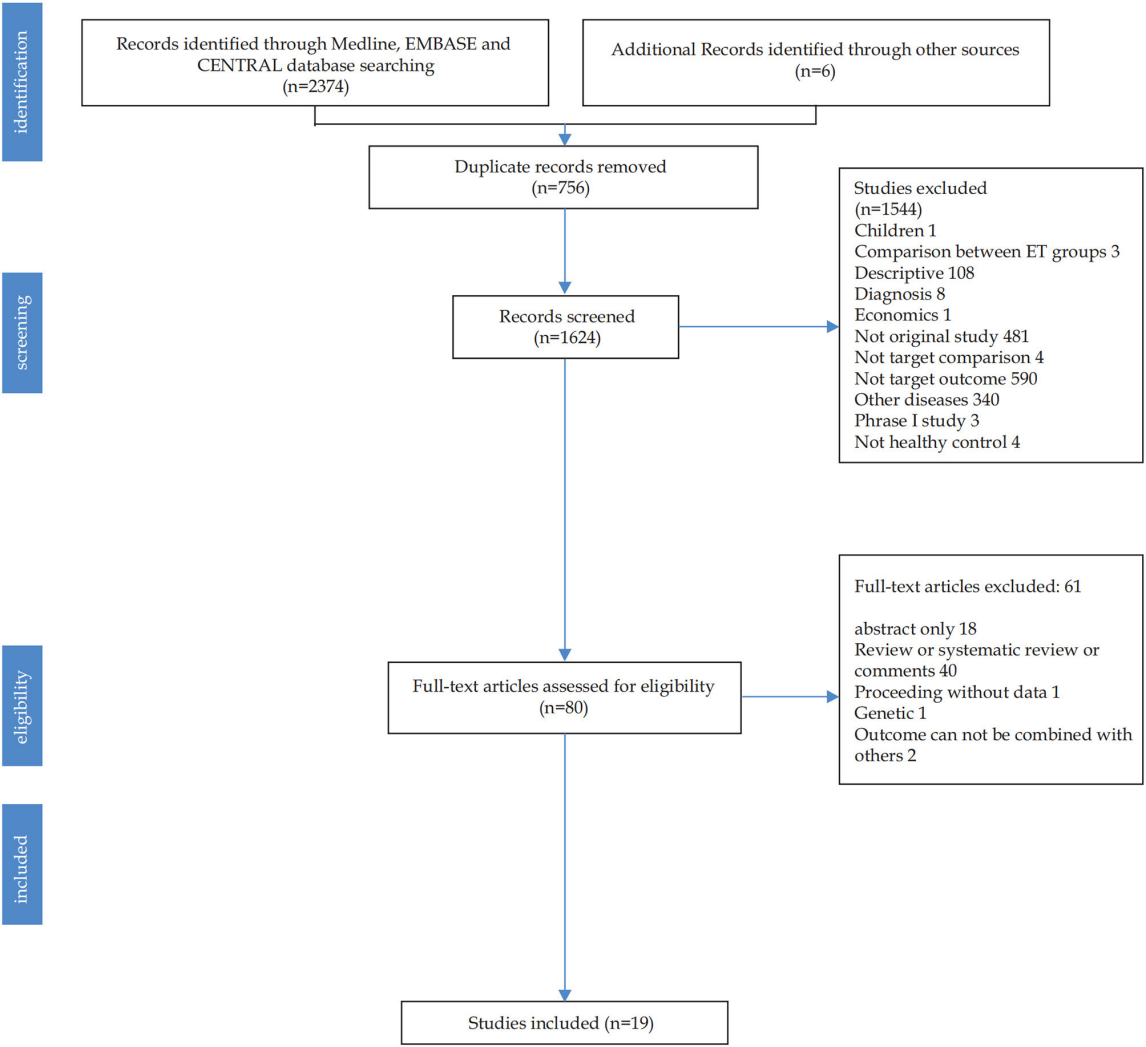
Flowchart of study selection.

Figure 1 shows the literature searching and study selection process. The initial search of databases retrieved 2,380 studies. After 756 duplicate records were removed, the remaining 80 studies with full text were read. Finally, 19 unique references were analyzed.

### Study Characteristics

The basic characteristics of the included studies are summarized in Table 1. All studies were conducted from different countries. Mean age varied from 27.7 to 84.4 years old. The average years of education were reported in 4 of 19 studies, varied from 3.4 to 16.6 years. Mean age at onset was reported in 5 of 19 studies, varying from 32.2 to 53.2 years old. Mean disease duration was described in 9 of 19 studies, varied from 3 to 16.6 years. Total 16 studies assessed cognitive function, 9 studies assessed depression or anxiety, and 1 study assessed mortality with patients with ET. Mean follow-up periods were reported in 8 of 19 studies, varied from 3.2 to 3.8 years. The 19 studies had different diagnostic criteria for ET, dementia, and affective disorders (Table 2).

**Table 2:** Characteristics of all ET patients.

**References**	**Year**	**Patient characteristics**
Dogu et al. (9)	2005	Based on the interview and examination, each neurologist independently assigned a diagnosis of ET or normal based on published diagnostic; criteria, which required the presence of moderate or greater amplitude upper limb kinetic tremor during ≥3 tests or an isolated head tremor.
Benito-León et al. (10)	2006	They had an action tremor of the head, limbs, or voice without any other recognizable cause; Second, the tremor had to be of gradual onset (i.e., slow and progressive) and present for at least 1 year or accompanied by a family history of the same disorder (at least one reportedly affected first degree relative). Third, on an Archimedes spiral, tremor severity had to be moderate or greater.
Louis et al. (11)	2007	Age >65 years; participants were diagnosed as having ET if they had an action tremor of the head or limbs without any other recognizable cause. The tremor had to be of gradual onset (i.e., slow and progressive) and either (i) present for at least 1 year or (ii) accompanied by a family history of the same disorder (at least one reportedly-affected first degree relative); dementia was diagnosed using criteria from the DSM-IV.
Louis et al. (12)	2007	Diagnostic criteria for ET, which were used both in participants who were examined and those whose medical records were reviewed, were closely modeled on those used in the Sicilian Study17and those recommended by Consensus from the Movement Disorders Society.
Kim et al. (13)	2009	All patients were diagnosed as having either definite or probable ET based on National Institutes of Health diagnostic criteria;
Thawani et al. (14)	2009	A total tremor score ≥5.5 or rated the handwritten sentence ≥2 (moderate or greater tremor, equivalent to a rating ≥5 in Bain and Findley); Based on neuropsychological testing, they demonstrated impairment in memory and at least 2 other cognitive domains, in the absence of delirium; Criteria for dementia from the DSM-III-R were applied in addition to ancillary information from medical charts and laboratory studies in the final evaluation.
Louis et al. (15)	2010	The diagnostic criteria for ET were those used in the Sicilian Study; participants were considered to have screened positive for dementia if: (1) they scored ≤ 23 points on the 37-MMSE; or (2) there were missing values;(i.e., participant failed to provide an answer) on the 37-MMSE; or (3) the participant or proxy provided information of a history of cognitive decline.
Louis et al. (16)	2010	Age ≥65 years; They was similar to the gender and education.
Passamonti et al. (18)	2011	Inclusion criteria for patients with essential tremor were: (i) integrity of the nigrostriatal dopaminergic terminals, as evidenced by a normal dopamine transporter scan, to exclude parkinsonisms; (ii) no traumatic brain injury and past or current substance abuse, particularly alcohol; (iii) no dementia according to the DSM-IV; in particular, probable Alzheimer's disease was excluded according to the NINCDS-ADRDA criteria.
Benito-León et al. (19)	2011	Diagnostic criteria for ET (used in participants who were examined and in those whose medical records were reviewed) were similar those used in the Sicilian Study; depressive symptoms were assessed with the simple question “Do you suffer from depression?”
Chandran et al. (20)	2012	All patients were diagnosed to have either definite or probable ET using the NIH Collaborative Genetic Criteria; HARS≥17; HDRS≥7.
Cerasa et al. (21)	2014	ET was diagnosed according to the consensus criteria of the Movement Disorders Society on tremor.
Rao et al. (22)	2013	ET participant using published diagnostic criteria (moderate or greater amplitude kinetic tremor during three or more activities, or a head tremor, in the absence of PD). Excluded participants with dementia (mMMSE score < 40), other neurological disorders (such as stroke, PD or dystonia), orthopedic impairments that impair walking, or depression. Diagnostic criteria for ET were similar to those used in the Sicilian study.
Benito-León et al. (23)	2013	The diagnosis of dementia was made by consensus of 2 neurologists, who applied the DSM-IV criteria. Depressive symptoms were assessed with the simple question “Do you suffer from depression?”
Benito-León et al. (23)	2015	All patients had no movement disorders other than ET, and they were diagnosed as either definite or probable ET based on the National Institutes of Health diagnostic criteria. dementia was diagnosed according to the criteria for dementia in the Diagnostic and DSM-IV.
Sengul et al. (25)	2016	ET diagnosed as per the Bain P. diagnostic criteria; The diagnostic criteria for ET were those used in the Sicilian Study;
Benito-León et al. (26)	2016	Participants were considered to have screened positive for dementia if: (1) they scored ≤ 23 points on the 37-MMSE; or (2) there were missing values; (i.e., participant failed to provide an answer) on the 37-MMSE; or (3) the participant or proxy provided information of a history of cognitive decline.
Meyers et al. (27)	2019	Diagnoses of ET were assigned based on published diagnostic criteria (moderate or greater amplitude kinetic tremor during three or more activities, or a head tremor in the absence of PD or another known cause [e.g., medication-induced tremor, tremor from hyperthyroidism; MoCA < 26.

*HARS, Hamilton Anxiety Rating Scale; HDRS, Hamilton Depression Rating Scale; mMMSE, modified Mini Mental State Examination; PD, Parkinson's disease; MoCA, Montreal Cognitive Assessment; DSM, The Diagnostic and Statistical Manual of Mental disorders*.

### Neuropsychological Assessment

The included studies relied on a diversity of neuropsychological assessments (Table 1). Ten studies used the MMSE (10, 13, 15, 16, 18, 21–24, 26), two studies used the MoCA (25, 27), one used the Hamilton Anxiety Scale (HAMA) and the BDI (18), one used the HDRS and the HARS (20), one used the Turkish versions of the HDRS and the Turkish versions of the HARS (9), six used the clinical assessment (11, 17–19, 23), and two used the Diagnostic and Statistical Manual of Mental disorders (DSM) (11, 14). These generalized screening instruments assessed global cognitive function, depression, and anxiety.

### Quantitative Analysis of Dementia

**Figure 2:**
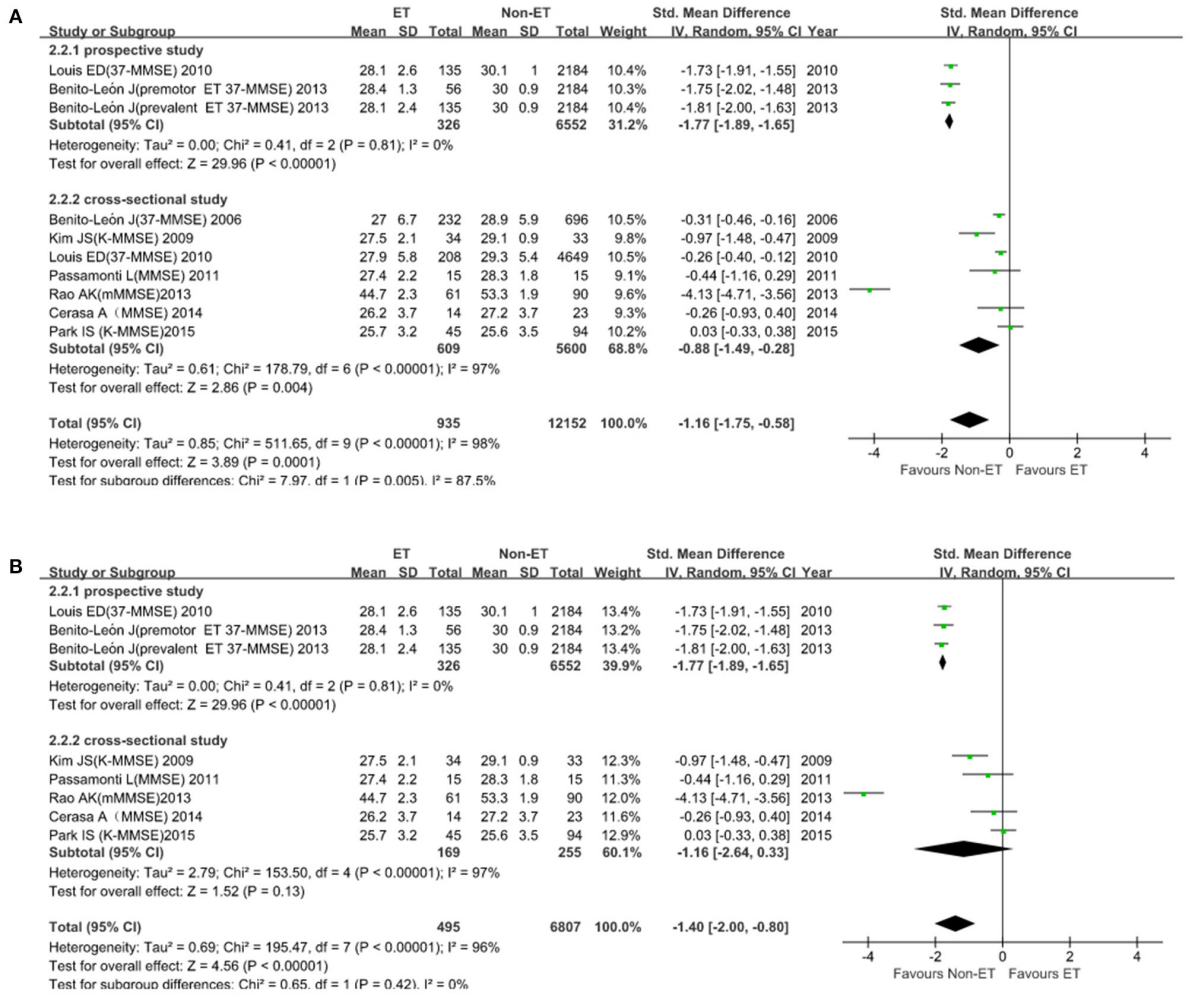
Mini-Mental State Examination (MMSE) score in patients between ET and Non-ET groups. **(A)**. Subgroup analyses of MMSE score between essential tremor (ET) and Non-ET. **(B)**. MMSE score between ET and Non-ET (2 studies were excluded).

Ten studies, i.e., 13,087 participants, were evaluated for MMSE. Heterogeneity across studies was high (*I*^2^ = 98%), so the random-effects model was chosen to analyze the results. In subgroup analysis, ET patients had significantly lower MMSE scores than that of Non-ET groups (SMD, −1.16; *95% CI*, −1.75 to −0.58; *p* = 0.0001; Figure 2A). After 2 studies were excluded for sensitivity analyses, the heterogeneity was lower (*I*^2^ = 0%) but the results remained significant (Figure 2B). There was no evidence of publication bias (Begg's test, *p* = 0.474).

**Figure 3:**
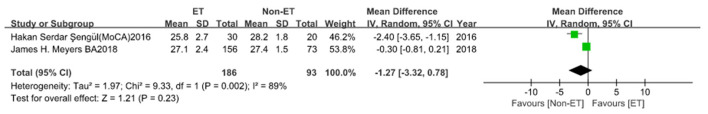
Montreal Cognitive Assessment (MoCA) score between essential tremor (ET) and Non-ET groups.

For MoCA scale (2 studies; 279 participants), the SMD was −0.52 (*95% CI*, −0.16 to 0.13; Figure 3). Heterogeneity across studies was high (*I*^2^ = 85%), so the random-effect model was adopted. ET was not associated with dementia (*p* = 0.23). There was no evidence of publication bias (Begg's test, *p* = 1.00).

### Quantitative Analysis of Affective Disorder

**Figure 4:**
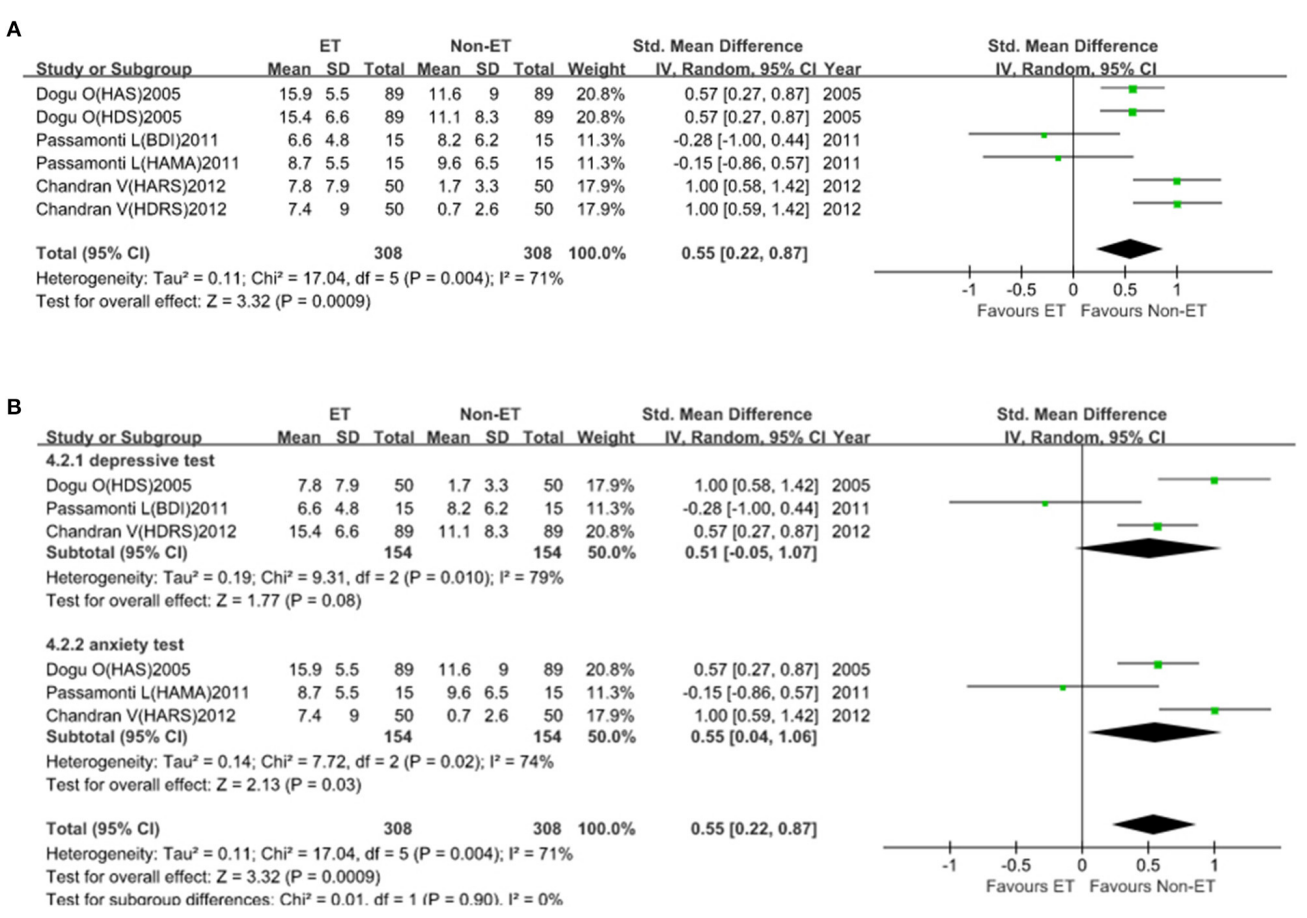
Depressive and anxiety symptoms scale score between essential tremor (ET) and Non-ET groups. **(A)**. The relationship between ET and depressive and anxiety symptom scale score. **(B)**. Subgroup analyses of the relationship between ET and depressive and anxiety symptom scale score.

For depressive and anxiety symptoms (6 studies with 616 participants), the SMD was 0.55 (95% *CI*, 0.22–0.87; Figure 4A). Heterogeneity across studies was high (*I*^2^ = 71%), so the random-effect model was adopted. In subgroup analysis, heterogeneity was lower (*I*^2^ = 0%) and patients with ET had significantly higher depressive and anxiety symptom scale score than that of Non-ET patients (*p* = 0.0009; Figure 4B). There was no evidence of publication bias (Begg's test, *p* = 0.707).

### Qualitative Analysis of Dementia and Affective Disorder

**Figure 5:**
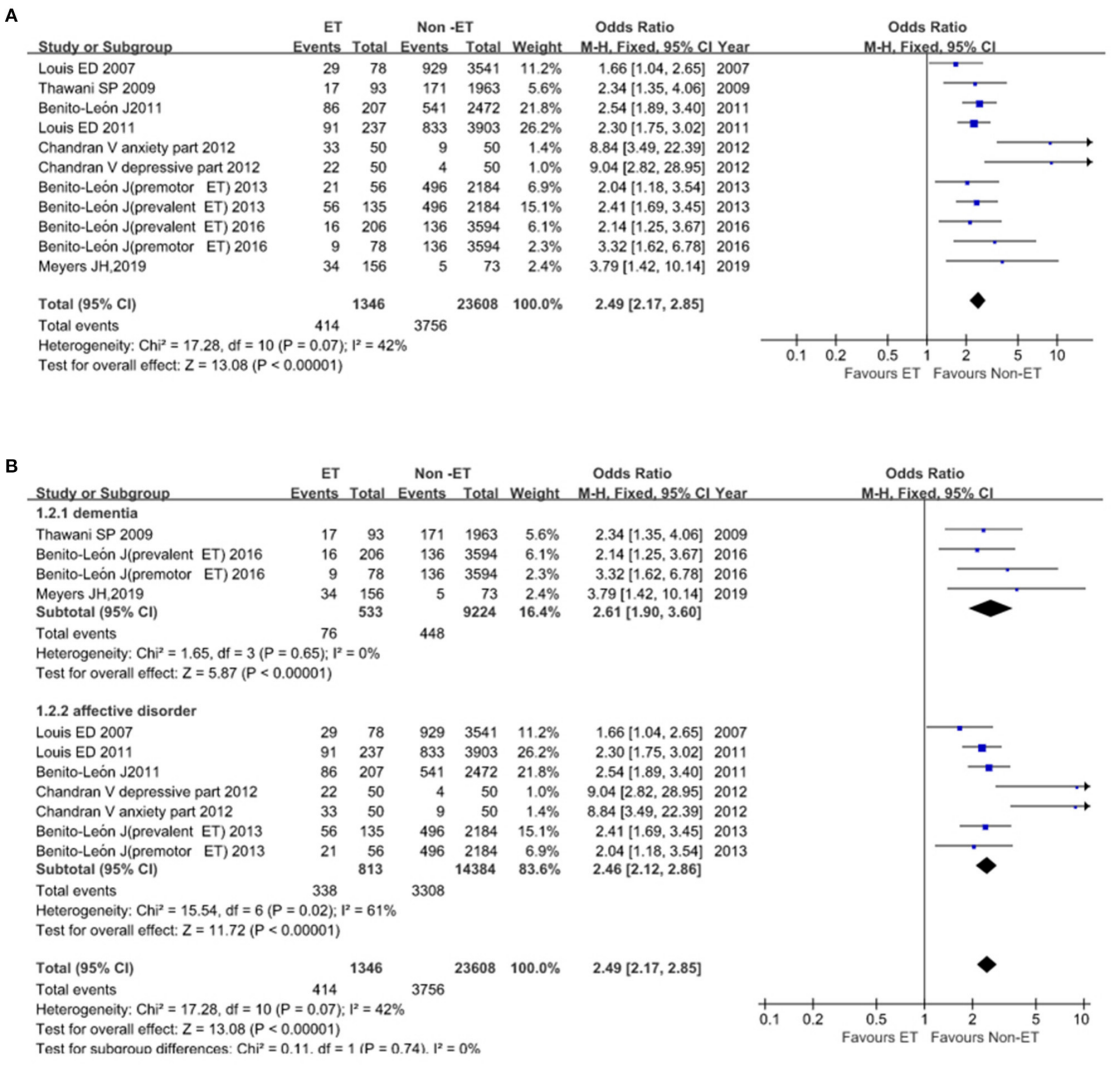
Association of dementia, affective disorders between essential tremor (ET) and Non-ET groups. **(A)**. The events of dementia and affective disorders in ET and Non-ET groups. **(B)**. Subgroup analyses of the events of dementia and affective disorders in ET and Non-ET groups.

Essential tremor was significantly associated with these two endpoints. Eleven studies that included 24,954 participants reported 4,170 events of dementia, affective disorders. Heterogeneity across studies was low to moderate (*I*^2^ = 42%; Figure 5A), the fixed-effect model was implemented. Heterogeneity was lower (*I*^2^ = 0%) in subgroup analysis and we found an increased risk of developing dementia, depression, and anxiety in the ET group compared with the Non-ET group (OR = 2.49, *95% CI*, 2.17–2.85, *p* < 0.00001; Figure 5B). There was no evidence of publication bias (Begg's test, *p* = 0.062).

### Quality of All Studies

For cross-sectional studies, the AHRQ scores varied from 5 to 7 (Table 3). For those prospective studies, the NOS scores varied from 6 to 8 stars (Table 4).

**Table 3:** Methodological quality assessments of included cross-sectional studies by the Agency for Healthcare Research and Quality (AHRQ).

**Question**	**Define the source of information**	**List inclusion and exclusion criteria for exposed and unexposed subjects (cases and controls) or refer to previous publications**	**Indicate time period used for identifying patients**	**Indicate whether or not subjects were consecutive if not population-based**	**Indicate if evaluators of subjective components of study were masked to other aspects of the status of the participants**	**Describe any assessments undertaken for quality assurance purposes**	**Explain any patient exclusions from analysis**	**Describe how confounding was assessed and/or controlled**.	**If applicable, explain how missing data were handled in the analysis**	**Summarize patient response rates and completeness of data collection**	**Clarify what follow-up, if any, was expected and the percentage of patients for which incomplete data or follow-up was obtained**	**Score**
**Answer**	Yes (+) or no /unclear (-)	Yes (+) or no /unclear (-)	Yes (+) or no /unclear (-)	Yes (+) or no /unclear (-)	Yes (+) or no /unclear (-)	Yes (+) or no /unclear (-)	Yes (+) or no /unclear (-)	Yes (+) or no /unclear (-)	Yes (+) or no /unclear (-)	Yes (+) or no /unclear (-)	Yes (+) or no /unclear (-)	
Dogu et al. (9)	**+**	**+**	**+**	**+**	**-**	**-**	**-**	**-**	**-**	**-**	**-**	**4**
Benito-León et al. (10)	**+**	**+**	**+**	**+**	**+**	**-**	**-**	**-**	**-**	**+**	**+**	**7**
Kim et al. (13)	**+**	**+**	**+**	**+**	**-**	**-**	**-**	**-**	**-**	**-**	**-**	**4**
Louis et al. (16)	**+**	**+**	**-**	**+**	**+**	**-**	**+**	**+**	**-**	**-**	**-**	**6**
Louis et al. (17)	**+**	**+**	**+**	**+**	**+**	**-**	**+**	**-**	**-**	**-**	**-**	**6**
Passamonti et al. (18)	**-**	**+**	**-**	**+**	**+**	**-**	**+**	**+**	**-**	**-**	**-**	**5**
Chandran et al. (20)	**+**	**+**	**+**	**+**	**+**	**+**	**-**	**-**	**-**	**-**	**-**	**6**
Cerasa et al. (21)	**+**	**+**	**+**	**+**	**+**	**-**	**-**	**-**	**-**	**-**	**-**	**5**
Rao et al. (22)	**+**	**+**	**-**	**+**	**+**	**+**	**+**	**-**	**-**	**-**	**-**	**6**
Park et al. (24)	**+**	**+**	**+**	**+**	**+**	**-**	**-**	**-**	**-**	**-**	**-**	**5**
Sengul et al. (25)	**+**	**+**	**+**	**+**	**+**	**-**	**-**	**-**	**-**	**-**	**-**	**5**
Meyers et al. (27)	**+**	**+**	**-**	**+**	**+**	**+**	**+**	**+**	**-**	**-**	**-**	**7**

**Table 4:** Methodological quality assessments of included observational studies by the Newcastle Ottawa Scale (NOS).

**References**	**Study design**	**Newcastle–ottawa scale**	
		**Selection**	**Comparability**	**Exposure**
Louis et al. (11)	Prospective cohort study	⋆⋆⋆⋆	⋆⋆	⋆⋆ 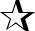
Louis et al. (12)	Prospective cohort study	⋆⋆⋆⋆	⋆⋆	⋆⋆⋆
Thawani et al. (14)	Prospective cohort study	⋆⋆⋆ 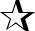	⋆⋆	⋆ 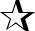 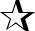
Louis et al. (15)	Prospective cohort study	⋆⋆⋆⋆	⋆⋆	⋆⋆ 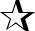
Benito-León et al. (19)	Prospective cohort study	⋆⋆⋆⋆	⋆⋆	⋆⋆ 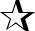
Benito-León et al. (23)	Prospective cohort study	⋆⋆⋆⋆	⋆⋆	⋆⋆ 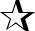
Benito-León et al. (26)	Prospective cohort study	⋆⋆⋆⋆	⋆⋆	⋆⋆ 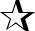

## Discussion

Essential tremor is characterized by a wide range of motor and non-motor symptoms. Patients with ET may exhibit dementia, depression, and anxiety. This meta-analysis indicated that there was a higher prevalence of dementia, depression, and anxiety in ET individuals not only in old age but also in the youth. The prevalence of ET among individuals aged between 18 and 60 was 1.60% (30). Recent studies have revealed that these non-motor features occur similarly in younger and elder patients (31). A study that included 45 young patients with ET and 35 age-matched controls showed that compared with the control group, depressive and anxiety symptoms were more common in the ET group, while total MoCA scores were lower in the ET group (32). This meta-analysis showed that the age of ET patients with cognitive and affective impairment ranged from 27 and 84 years old. Therefore, non-motor symptoms seem to be disease-associated rather than age-associated manifestations. ET appears to be a risk factor for developing dementia, depression, and anxiety. A clinical study demonstrated that Chinese patients with ET showed a high prevalence of depression and anxiety, suggesting that depression and anxiety were risk factors for dementia (33). In a prospective population-based study, elderly-onset ET was associated with an increased risk of dementia (34). Our study showed that the OR for dementia, depression, and anxiety in individuals of the ET group was 2.49-fold than that of Non-ET group. Therefore, ET was speculated as an independent factor of dementia and affective disorders. Early screening and intervention for dementia, depression, and anxiety in patients with ET should be a crucial strategy to delay cognitive or affective impairment progression.

Our meta-analysis also revealed that the all-cause mortality rate in the ET group was higher than that of the control group. However, whether dementia, depression, or anxiety are risk factors for all-cause death in the ET group remains unclear. For Parkinson's disease (PD), several studies examined the extent to which cognitive impairment increased the risk of death (35–42). Eight studies included in this meta-analysis had a follow-up of 3.2–3.8 years. Among them, only one reported the mortality rates in ET and Non-ET groups. To find out whether cognitive or affective dysfunctions are the risk factors of all-cause mortality in ET patients, future studies with more participants and longer follow-up are needed.

In the general population of non-demented individuals, the predictors of cognitive change include demographic, genetic, medical, subjective cognitive, and biologic factors (30, 43–50). According to previous studies about PD, the predictors of cognitive impairment include a distinctive set of cognitive, neurologic, psychiatric, biologic, and genetic factors that may be disease-specific and factors related to the clinical severity of PD (51). Understanding the predictors of cognitive and emotional changes will be helpful for early screening, timely intervention, and improvement of quality of life with ET patients. In our study, we could not analyze the predictors of cognitive and affective disorders due to the lack of data from the multiple-factor analysis. In the future, prospective studies are needed to explore predictors.

The cognitive impairment of patients with ET mainly affected the executive function. It is evident that the cerebellum is responsible for executive function and emotion-related behaviors (52, 53). Executive deficits in patients with ET have generally been attributed to the inefficient cerebellar-cortical networks, particularly, those projecting to and from the prefrontal cortex (53–59). A possible link between Lewy body dementia, and/or PD and ET has been reported (60). One neuroimaging study has shown that the white matter and gray matter abnormalities significantly correlated with tremor severity, cognitive profile, and depression (61). Furthermore, a study reported the correlation between brain microstructural changes in the amygdala/left ventrolateral prefrontal cortex and the severity of depressive/anxiety symptoms among patients with ET (62). Therefore, dynamic assessment of dementia, depression, and anxiety and neuroimaging with ET patients will help to explain the mechanism of the occurrence and development of these diseases.

Several limitations of our study should be noted. Firstly, different evaluation tools were used in the studies to assess cognitive function, depression, and anxiety, which may cause high heterogeneity in this meta-analysis. Secondly, most of these studies were cross-sectional ones and patient information was incomplete. Since both outcomes and exposures are ascertained at the same time, the temporal relationship between the two might be unclear (63). Thirdly, our meta-analysis is based on observational studies, which compared ET patients and Non-ET controls. In future studies, uniform diagnosis and evaluation criteria are needed in prospective studies to better understand the association of ET with dementia, depression, and anxiety.

In conclusion, we found a higher prevalence of dementia, depression, and anxiety in the ET group compared with the Non-ET group. Therefore, non-motor symptoms should not be neglected among patients with ET. However, the causal relationship between ET and dementia, depression, and anxiety is unclear and should be further investigated.

## Data Availability Statement

The original contributions presented in the study are included in the article/Supplementary Material, further inquiries can be directed to the corresponding authors.

## Author Contributions

YS, XC, MA, XG, SD, MZ, CY, LW, JZ, and LZ: collected and analyzed data. TB and XL: designed and supervised the study. All authors contributed to the article and approved the submitted version.

## Funding

This work was supported by the National Science Foundation of China (Grant Nos. 82160272 to XL and 81873354), Research Program of Yunnan Provincial Health Commission (Grant No. D-2019013 to XL), Yunnan Province Clinical Research Center for Neurological Diseases (Grant No. 202102AA100061 to LZ), Yunnan Education Program (Grant No. SYSX202036 to XL), Research Program of Yunnan Science and Technology Department (Grant Nos. 2019FE001(-222), 2019FE001(-107), 202101AT070151 to XL, and 202101AY070001-048), and the Spring City-Kunming Young Top Talent Research Fund Project (C201914016).

## Conflict of Interest

JZ was employed by Bothwin Clinical Study Consultant. The remaining authors declare that the research was conducted in the absence of any commercial or financial relationships that could be construed as a potential conflict of interest.

## Publisher's Note

All claims expressed in this article are solely those of the authors and do not necessarily represent those of their affiliated organizations, or those of the publisher, the editors and the reviewers. Any product that may be evaluated in this article, or claim that may be made by its manufacturer, is not guaranteed or endorsed by the publisher.

## References

[1] Louis ED Ford B Barnes LF. Clinical subtypes of essential tremor. Arch Neurol. (2000) 57:1194–8. 10.1001/archneur.57.8.1194 10927801

[2] Bhatia KP Bain P Bajaj N Elble RJ Hallett M Louis ED. Consensus statement on the classification of tremors from the task force on tremor of the international parkinson and movement disorder society. Mov Disord. (2018) 33:75–87. 10.1002/mds.27121 29193359 PMC6530552

[3] Bermejo-Pareja F. Essential tremor–a neurodegenerative disorder associated with cognitive defects? Nat Rev Neurol. (2011) 7:273–82. 10.1038/nrneurol.2011.44 21487422

[4] Jiménez-Jiménez FJ Alonso-Navarro H García-Martín E Agúndez JAG. Sleep disorders in patients with essential tremor. Curr Neurol Neurosci Rep. (2021) 21:23. 10.1007/s11910-021-01109-y 33754217

[5] Jiménez-Jiménez FJ Alonso-Navarro H García-Martín E Agúndez JAG. Sleep disorders in essential tremor: systematic review and meta-analysis. Sleep. (2020) 43:zsaa039. 10.1093/sleep/zsaa039 32163585

[6] Bermejo-Pareja F Puertas-Martin V. Cognitive features of essential tremor: a review of the clinical aspects and possible mechanistic underpinnings. Tremor Other Hyperkinet Mov. (2012) 2:02-74-541-1. 10.7916/D89W0D7W 23440004 PMC3572680

[7] Sinoff G Badarny S. Mild cognitive impairment, dementia, and affective disorders in essential tremor: a prospective study. Tremor Other Hyperkinet Mov. (2014) 4:227. 10.5334/tohm.179 25009763 PMC4069695

[8] Stroup DF Berlin JA Morton SC Olkin I Williamson GD Rennie D. Meta-analysis of observational studies in epidemiology: a proposal for reporting. Meta-analysis of observational studies in epidemiology (MOOSE) group. JAMA. (2000) 283:2008–12. 10.1001/jama.283.15.2008 10789670

[9] Dogu O Louis ED Sevim S Kaleagasi H Aral M. Clinical characteristics of essential tremor in Mersin, Turkey–a population-based door-to-door study. J Neurol. (2005) 252:570–4. 10.1007/s00415-005-0700-8 15778813

[10] Benito-León J Louis ED Bermejo-Pareja F. Population-based case-control study of cognitive function in essential tremor. Neurology. (2006) 66:69–74. 10.1212/01.wnl.0000192393.05850.ec 16401849

[11] Louis ED Benito-León J Bermejo-Pareja F. Self-reported depression and anti-depressant medication use in essential tremor: cross-sectional and prospective analyses in a population-based study. Eur J Neurol. (2007) 14:1138–46. 10.1111/j.1468-1331.2007.01923.x 17708753

[12] Louis ED Benito-León J Ottman R Bermejo-Pareja F. A population-based study of mortality in essential tremor. Neurology. (2007) 69:1982–9. 10.1212/01.wnl.0000279339.87987.d7 18025392

[13] Kim JS Song IU Shim YS Park JW Yoo JY Kim YI. Cognitive impairment in essential tremor without dementia. J Clin Neurol. (2009) 5:81–4. 10.3988/jcn.2009.5.2.81 19587814 PMC2706415

[14] Thawani SP Schupf N Louis ED. Essential tremor is associated with dementia: prospective population-based study in New York. Neurology. (2009) 73:621–5. 10.1212/WNL.0b013e3181b389f1 19704081 PMC2731620

[15] Louis ED Benito-León J Vega-Quiroga S Bermejo-Pareja F. Faster rate of cognitive decline in essential tremor cases than controls: a prospective study. Eur J Neurol. (2010) 17:1291–7. 10.1111/j.1468-1331.2010.03122.x 20561042 PMC2939209

[16] Louis ED Benito-León J Vega-Quiroga S Bermejo-Pareja F. Cognitive and motor functional activity in non-demented community-dwelling essential tremor cases. J Neurol Neurosurg Psychiatry. (2010) 81:997–1001. 10.1136/jnnp.2009.202838 20547612

[17] Louis ED Benito-León J Vega S Bermejo-Pareja F. Frailty in elderly persons with essential tremor: a population-based study (NEDICES). Eur J Neurol. (2011) 18:1251–7. 10.1111/j.1468-1331.2011.03374.x 21426443 PMC3135673

[18] Passamonti L Novellino F Cerasa A Chiriaco C Rocca F Matina MS. Altered cortical-cerebellar circuits during verbal working memory in essential tremor. Brain. (2011) 134:2274–86. 10.1093/brain/awr164 21747127

[19] Benito-León J Louis ED Mitchell AJ Bermejo-Pareja F. Elderly-onset essential tremor and mild cognitive impairment: a population-based study (NEDICES). J Alzheimers Dis. (2011) 23:727–35. 10.3233/JAD-2011-101572 21304183

[20] Chandran V Pal PK Reddy JY Thennarasu K Yadav R Shivashankar N. Non-motor features in essential tremor. Acta Neurol Scand. (2012) 125:332–7. 10.1111/j.1600-0404.2011.01573.x 21777207

[21] Cerasa A Nisticò R Salsone M Bono F Salvino D Morelli M„. Neuroanatomical correlates of dystonic tremor: a cross-sectional study. Parkinsonism Relat Disord. (2014) 20:314–7. 10.1016/j.parkreldis.2013.12.007 24405756

[22] Rao AK Uddin J Gillman A Louis ED. Cognitive motor interference during dual-task gait in essential tremor. Gait Posture. (2013) 38:403–9. 10.1016/j.gaitpost.2013.01.006 23369662 PMC3679258

[23] Benito-León J Louis ED Sánchez-Ferro Á Bermejo-Pareja F. Rate of cognitive decline during the premotor phase of essential tremor: a prospective study. Neurology. (2013) 81:60–6. 10.1212/WNL.0b013e318297ef2b 23700331 PMC3770204

[24] Park IS Oh YS Lee KS Yang DW Song IU Park JW. Subtype of mild cognitive impairment in elderly patients with essential tremor. Alzheimer Dis Assoc Disord. (2015) 29:141–5. 10.1097/WAD.0000000000000054 25037029

[25] Sengul HS Sengul Y Yucel S Forta H. Cognitive impairment in young multiple sclerosis and essential tremor patients: a comparative study. Turk Noroloji Dergisi. (2016) 22:109–13. 10.4274/tnd.46704

[26] Benito-León J Contador I Louis ED Cosentino S Bermejo-Pareja F. Education and risk of incident dementia during the premotor and motor phases of essential tremor (NEDICES). Medicine. (2016) 95:e4607. 10.1097/MD.0000000000004607 27537597 PMC5370823

[27] Meyers JH Hickman R Cristal AD Factor-Litvak P Cosentino S Louis ED. More unaffected first-degree relatives of essential tremor cases have mild cognitive deficits than age-matched controls. Parkinsonism Relat Disord. (2019) 61:144–50. 10.1016/j.parkreldis.2018.10.030 30404762 PMC6488412

[28] Stang A. Critical evaluation of the Newcastle-Ottawa scale for the assessment of the quality of nonrandomized studies in meta-analyses. Eur J Epidemiol. (2010) 25:603–5. 10.1007/s10654-010-9491-z 20652370

[29] Patsopoulos NA Evangelou E Ioannidis JP. Heterogeneous views on heterogeneity. Int J Epidemiol. (2009) 38:1740–2. 10.1093/ije/dyn235 18940836 PMC4719167

[30] Rabin JS Schultz AP Hedden T Viswanathan A Marshall GA Kilpatrick E. Interactive associations of vascular risk and β-amyloid burden with cognitive decline in clinically normal elderly individuals: findings from the harvard aging brain study. JAMA Neurol. (2018) 75:1124–31. 10.1001/jamaneurol.2018.1123 29799986 PMC6143121

[31] Shalash AS Mohamed H Mansour AH Elkady A Elrassas H Hamid E. Clinical profile of non-motor symptoms in patients with essential tremor: impact on quality of life and age-related differences. Tremor Other Hyperkinet Mov. (2019) 9. 10.7916/tohm.v0.736 31867132 PMC6898893

[32] Sengul Y Sengul HS Yucekaya SK Yucel S Bakim B Pazarci NK. Cognitive functions, fatigue, depression, anxiety, and sleep disturbances: assessment of nonmotor features in young patients with essential tremor. Acta Neurol Belg. (2015) 115:281–7. 10.1007/s13760-014-0396-6 25471376

[33] Huang H Yang X Zhao Q Chen Y Ning P Shen Q. Prevalence and risk factors of depression and anxiety in essential tremor patients: a cross-sectional study in Southwest China. Front Neurol. (2019) 10:1194. 10.3389/fneur.2019.01194 31803131 PMC6873801

[34] Bermejo-Pareja F Louis ED Benito-León J. Risk of incident dementia in essential tremor: a population-based study. Mov Disord. (2007) 22:1573–80. 10.1002/mds.21553 17516478

[35] Bugalho P Ladeira F Barbosa R Marto JP Borbinha C Salavisa M. Motor and non-motor function predictors of mortality in Parkinson's disease. J Neural Transm. (2019) 126:1409–15. 10.1007/s00702-019-02055-3 31385098

[36] Auyeung M Tsoi TH Mok V Cheung CM Lee CN Li R. Ten year survival and outcomes in a prospective cohort of new onset Chinese Parkinson's disease patients. J Neurol Neurosurg Psychiatry. (2012) 83:607–11. 10.1136/jnnp-2011-301590 22362919

[37] Hobson P Meara J. Mortality and quality of death certification in a cohort of patients with Parkinson's disease and matched controls in North Wales, UK at 18 years: a community-based cohort study. BMJ Open. (2018) 8:e018969. 10.1136/bmjopen-2017-018969 29444783 PMC5829780

[38] Hely MA Morris JG Traficante R Reid WG O'Sullivan DJ Williamson PM. The sydney multicentre study of Parkinson's disease: progression and mortality at 10 years. J Neurol Neurosurg Psychiatry. (1999) 67:300–7. 10.1136/jnnp.67.3.300 10449550 PMC1736543

[39] Pinter B Diem-Zangerl A Wenning GK Scherfler C Oberaigner W Seppi K. Mortality in Parkinson's disease: a 38-year follow-up study. Mov Disord. (2015) 30:266–9. 10.1002/mds.26060 25447933

[40] Bäckström D Granåsen G Domellöf ME Linder J Jakobson Mo S. Early predictors of mortality in parkinsonism and Parkinson disease: a population-based study. Neurology. (2018) 91:e2045–56. 10.1212/WNL.0000000000006576 30381367 PMC6282235

[41] Manole E Dumitrescu L Niculi?e C Popescu BO Ceafalan LC. Potential roles of functional bacterial amyloid proteins, bacterial biosurfactants and other putative gut microbiota products in the etiopathogeny of Parkinson's Disease. Biocell. (2021) 45:1–16. 10.32604/biocell.2021.013452

[42] Beyer MK Herlofson K Arsland D Larsen JP. Causes of death in a community-based study of Parkinson's disease. Acta Neurol Scand. (2001) 103:7–11. 10.1034/j.1600-0404.2001.00191.x 11153892

[43] Vemuri P Lesnick TG Przybelski SA Knopman DS Preboske GM Kantarci K. Vascular and amyloid pathologies are independent predictors of cognitive decline in normal elderly. Brain. (2015) 138:761–71. 10.1093/brain/awu393 25595145 PMC4339775

[44] Adak S Illouz K Gorman W Tandon R Zimmerman EA Guariglia R. Predicting the rate of cognitive decline in aging and early Alzheimer disease. Neurology. (2004) 63:108–14. 10.1212/01.WNL.0000132520.69612.AB 15249619

[45] Oh H Madison C Haight TJ Markley C Jagust WJ. Effects of age and β-amyloid on cognitive changes in normal elderly people. Neurobiol Aging. (2012) 33:2746–55. 10.1016/j.neurobiolaging.2012.02.008 22429886 PMC3381075

[46] Dik MG Jonker C Comijs HC Bouter LM Twisk JW van Kamp GJ. Memory complaints and APOE-epsilon4 accelerate cognitive decline in cognitively normal elderly. Neurology. (2001) 57:2217–22. 10.1212/WNL.57.12.2217 11756600

[47] Teunissen CE Blom AH Van Boxtel MP Bosma H de Bruijn C Jolles J. Homocysteine: a marker for cognitive performance? a longitudinal follow-up study. J Nutr Health Aging. (2003) 7:153–9. 12766792

[48] Fiocco AJ Lindquist K Ferrell R Li R Simonsick EM Nalls M. COMT genotype and cognitive function: an 8-year longitudinal study in white and black elders. Neurology. (2010) 74:1296–302. 10.1212/WNL.0b013e3181d9edba 20404311 PMC2860484

[49] Van Gerven PW Van Boxtel MP Ausems EE Bekers O Jolles J. Do apolipoprotein E genotype and educational attainment predict the rate of cognitive decline in normal aging? a 12-year follow-up of the maastricht aging study. Neuropsychology. (2012) 26:459–72. 10.1037/a0028685 22642392

[50] Tupler LA Krishnan KR Greenberg DL Marcovina sm Payne ME MacFall JR. Predicting memory decline in normal elderly: genetics, MRI, and cognitive reserve. Neurobiol Aging. (2007) 28:1644–56. 10.1016/j.neurobiolaging.2006.07.001 16916565

[51] Louis ED Joyce JL Cosentino S. Mind the gaps: what we don't know about cognitive impairment in essential tremor. Parkinsonism Relat Disord. (2019) 63:10–9. 10.1016/j.parkreldis.2019.02.038 30876840 PMC6682425

[52] Thangavelu K Talk AC Clark GI Dissanayaka NNW. Psychosocial factors and perceived tremor disability in essential tremor. Neurosci Biobehav Rev. (2020) 108:246–53. 10.1016/j.neubiorev.2019.10.021 31682885

[53] Koziol LF Budding D Andreasen N D'Arrigo S Bulgheroni S Imamizu H. Consensus paper: the cerebellum's role in movement and cognition. Cerebellum. (2014) 13:151–77. 10.1007/s12311-013-0511-x 23996631 PMC4089997

[54] Schmahmann JD Sherman JC. The cerebellar cognitive affective syndrome. Brain. (1998) 121 (Pt 4):561–79. 10.1093/brain/121.4.561 9577385

[55] Akshoomoff NA Courchesne E Townsend J. Attention coordination and anticipatory control. Int Rev Neurobiol. (1997) 41:575–98. 10.1016/S0074-7742(08)60371-2 9378609

[56] Hallett M Grafman J. Executive function and motor skill learning. Int Rev Neurobiol. (1997) 41:297–323. 10.1016/S0074-7742(08)60357-8 9378593

[57] Li J Zhang Y Zhang T Tian M Hou J Huang D. Analysis of isolation of cerebral cortical neurons in rats by different methods. Biocell. (2020) 44:209–15. 10.32604/biocell.2020.08941

[58] Desmond JE Fiez JA. Neuroimaging studies of the cerebellum: language, learning and memory. Trends Cogn Sci. (1998) 2:355–62. 10.1016/S1364-6613(98)01211-X 21227232

[59] Rapoport M van Reekum R Mayberg H. The role of the cerebellum in cognition and behavior: a selective review. J Neuropsychiatry Clin Neurosci. (2000) 12:193–8. 10.1176/jnp.12.2.193 11001597

[60] Louis ED Benito-Leon J Faust PL. Essential tremor seems to be a risk factor for Parkinson's disease. Parkinsonism Relat Disord. (2016) 26:82–3. 10.1016/j.parkreldis.2016.02.026 26972525

[61] Pietracupa S Bologna M Bharti K Pasqua G Tommasin S Elifani F. White matter rather than gray matter damage characterizes essential tremor. Eur Radiol. (2019) 29:6634–42. 10.1007/s00330-019-06267-9 31139970

[62] Sengul Y Otcu H Ustun I Sengul HS Cersonsky T Alkan A. Neuroimaging depression and anxiety in essential tremor: a diffusion tensor imaging study. Clin Imaging. (2019) 58:96–104. 10.1016/j.clinimag.2019.06.016 31284179

[63] Grimes DA Schulz KF. An overview of clinical research: the lay of the land. Lancet. (2002) 359:57–61. 10.1016/S0140-6736(02)07283-5 11809203

